# Nutrient Availability Alters the Effect of Autophagy on Sulindac Sulfide-Induced Colon Cancer Cell Apoptosis

**DOI:** 10.1155/2012/897678

**Published:** 2012-12-09

**Authors:** Shiun-Kwei Chiou, Neil Hoa, Lisheng Ge, Martin R. Jadus

**Affiliations:** ^1^Research Division, Veterans Affairs Medical Center, 5901 E 7th Street, Long Beach, CA 90822, USA; ^2^Department of Medicine, University of California, 101 The City Drive, Irvine, Orange, CA 92868, USA; ^3^UCI Pathology Department, Chao Family Comprehensive Cancer Center, University of California, Irvine 92697, USA; ^4^Neuro-Oncology Program, Chao Comprehensive Cancer Center, UC Irvine School of Medicine, University of California, Irvine, Orange, CA 92868, USA

## Abstract

Autophagy is a catabolic process by which a cell degrades its intracellular materials to replenish itself. Induction of autophagy under various cellular stress stimuli can lead to either cell survival or cell death via apoptotic and/or autophagic (nonapoptotic) pathways. The NSAID sulindac sulfide induces apoptosis in colon cancer cells. Here, we show that inhibition of autophagy under serum-deprived conditions resulted in significant reductions of sulindac sulfide-induced apoptosis in HT-29 colon cancer cells. In contrast, inhibition of autophagy under conditions where serum is available significantly increased sulindac sulfide-induced apoptosis in HT-29 cells. We previously showed that the apoptosis inhibitor, survivin, plays a role in regulating NSAID-induced apoptosis and autophagic cell death. Here, we show that survivin protein half-life is increased in the presence of autophagy inhibitors under serum-deprived conditions, but not under conditions when serum is available. Thus, the increased levels of survivin may be a factor contributing to inhibition of sulindac sulfide-induced apoptosis under serum-deprived conditions. These results suggest that whether a cell lives or dies due to autophagy induction depends on the balance of factors that regulate both autophagic and apoptotic processes.

## 1. Introduction

Autophagy is an evolutionarily conserved process that breaks down damaged/unwanted cellular materials, such as organelles, protein aggregates, and macromolecules, into amino acids to be recycled by the cell for its metabolic and survival needs [1, 2]. It is active in all cells and inducible by a number of cellular stresses, including nutrient deprivation and oxidative stress. Autophagy is initiated by the formation of double-membrane vacuoles, autophagosomes, that quarantine the cytosolic materials and subsequently fuses with the lysosomes to degrade its contents. Autophagosome formation requires cleavage of microtubule-associated protein light chain 3b (LC3b) by Atg 4 to form cytosolic LC3b-I [3]. LC3b-I undergoes conjugation to a lipid moiety, by the Atg 7 and Atg 3 enzymes, to generate LC3b-II, which incorporates into the autophagosome membrane with aid from the Atg12-Apg 5-Atg16 protein complex [4, 5]. Together, the entire complex is also responsible for the elongation and curvature of the vacuole to generate a mature autophagosome [6, 7]. Detection of LC3b-II by western blotting is a commonly utilized and widely accepted assay for monitoring autophagy induction. Degradation of proteins that are known to be degraded through the autophagy process, such as p62, is utilized to monitor the autophagic flux [8]. In this study, these detection methods were utilized to monitor autophagy and its inhibition.

Although the prosurvival function of autophagy is well known and defined, recent studies indicate that autophagy induction may also trigger apoptosis-independent cell death, or “autophagic cell death.” At present, it is unclear when and where autophagy is prosurvival, and when and where it is prodeath [9, 10]. Autophagy is also reported to influence the cell's susceptibility to apoptosis. The connection between autophagy and apoptosis is poorly understood and currently is an area of intense research. A number of studies indicate that autophagy can ensure cell survival by inhibiting apoptosis (e.g., reviewed in [11, 12]). However, some recent studies indicate that autophagy sometimes leads to and is associated with apoptosis (e.g., [13, 14]). 

Sulindac sulfide, a nonsteroidal anti-inflammatory drug (NSAID), possesses proapoptotic, anticancer, and anti-inflammatory activities. Sulindac sulfide induces apoptosis in gastrointestinal cancer cells. The inhibition of autophagy in colon cancer HT-29 cells is reported to sensitize these cells to sulindac sulfide-induced apoptosis [15]. Conversely, our recent study in gastric cancer AGS cells showed that inhibition of autophagy is associated with inhibition of survivin down-regulation by sulindac sulfide that resulted in increased apoptosis [16]. How and why autophagy is proapoptosis in one setting and antiapoptosis in another is unclear. Here, we investigated the effect of autophagy on the apoptotic response of HT-29 cells to sulindac sulfide in different contexts: under serum-deprivation and normal serum conditions. We also examined the role of survivin in apoptosis under these conditions. 

## 2. Materials and Methods

### 2.1. Cell Culture and Treatments

HT-29 was cultured in McCoy's 5A modified medium (ATCC, Manassas, VA, USA) supplemented with 10% fetal bovine serum (Atlanta Biologicals, Lawrenceville, GA, USA) and 1% antibiotics (Penicillin G, Amphotericin B and Streptomycin; Mediatech Cellgro, Hernodon, VA, USA). For all assays, cells were treated with 0.3 mM sulindac sulfide. This high concentration of sulindac sulfide was utilized because cells in the colonic mucosa are normally exposed to high concentrations of NSAIDs. Thus, this concentration better represents the physiological value.

### 2.2. siRNA Transfections

A commercially available pool of 3 target-specific siRNAs designed to silence human Atg7 (Santa Cruz Biotechnologies, Santa Cruz, CA, USA) was utilized. 10 nM of siRNAs was transfected into HT-29 cells at 70% confluence using RNAiMax transfection reagent (Invitrogen, Carlsbad, CA, USA), following the manufacturer's instructions. Mock transfection utilizing only the transfection reagent without any siRNA, and transfection of 10 nM of a commercially available short RNA (Santa Cruz Biotechnologies, Santa Cruz, CA, USA) that does not silence mammalian mRNAs were included as controls. The nonsilencing control RNA is conjugated to a green fluorescent dye for monitoring transfection efficiency with fluorescence microscopy. The percent of fluorescent cells per 500 total cells were counted to determine the transfection efficiency. At 24 hours after transfection: (1) for the autophagy inhibition assay, cells were incubated in serum-containing and serum-free media for the durations indicated in figures. At these times cell lysates were collected for western blot analysis of LC3*β* II and beta actin levels, and for p62 and beta actin levels in the presence of 100 *μ*g/mL cycloheximide (CHX) ± 10 mM 3-MA; (2) for the apoptosis assays, cells were incubated in serum free media overnight, then treated with 0.3 mM sulindac sulfide for the indicated durations, and in media containing 5% FBS overnight, and then treated with 0.3 mM sulindac sulfide for the indicated durations. Fold apoptosis was determined as described below; (3) For the survivin half-life determination, cells were incubated in serum free media and media containing 5% FBS overnight, and treated as described below; (4) for survivin mRNA expression assays, cells were incubated in serum-free media and media containing 5% FBS for the durations indicated, and total RNA and real-time quantitative PCR was performed as described below.

### 2.3. Apoptosis Assays

Equal numbers of HT-29 cells were seeded in each well of 6-well culture plates and allowed to attach overnight. Then cells were incubated overnight in media containing 5% FBS and media containing no serum. Vehicle (DMSO) and sulindac sulfide were added to the media for the durations indicated in figure. Apoptosis was determined by comparing the fold caspase-3 activation between samples containing equal numbers of HT-29 cells. The colorimetric CaspACE assay system (Promega, Madison, WI, USA), was utilized for measuring caspase-3 activity, following the manufacturer's instructions. The annexin V binding assay kit (Abcam Inc., Cambridge, MA, USA) was also utilized to measure apoptosis induction, following the manufacturer's instructions. % apoptosis was determined by counting the percentage of fluorescent-labeled (annexin V bound) cells in a total of 500 cells. 

### 2.4. Western Blot Analysis and Protein Signal Quantification

Cells lysates were prepared as described previously [17]. 150 *μ*g protein per sample was separated by 12% SDS-PAGE and transferred onto nitrocellulose membranes. The membranes were blocked in skim milk and incubated with rabbit-polyclonal anti-LC3*β* antibodies, rabbit polyclonal anti-survivin antibodies (both Novus Biologicals, Littleton, CO, USA), rabbit polyclonal anti-p62 antibodies and rabbit polyclonal anti-Atg7 antibodies (both Santa Cruz Biotechnologies, Santa Cruz, CA, USA) at 4°C overnight. The anti-LC3*β* antibody utilized in these assays recognizes both the LC3*β* I and LC3*β* II forms. This antibody was used in order to confirm that LC3*β* I was present in the samples, and that LC3*β* II conversion was observed only under conditions when autophagy was induced. The membrane was then washed and incubated for 1 hour with peroxidase conjugated goat-anti-rabbit secondary antibodies (Sigma, St. Louis, MO, USA). The same membrane was then stripped with Re-blot Mild solution (Millipore, Temecula, CA, USA) according to the manufacturer's instructions, and then incubated with mouse monoclonal anti-beta-actin antibodies for 1 hour, washed, and incubated for 1 hour with peroxidase conjugated goat-anti-mouse secondary antibodies (Sigma, St. Louis, MO, USA). Protein signals were measured utilizing densitometry software, and normalized to beta-actin levels, as described previously [17]. All protein signal data were averaged from three repeated experiments.

### 2.5. RNA Isolation, Reverse Transcription, and Real-Time Quantitative PCR Analysis

Total RNA was isolated from HT-29 cells using the RNeasy mini kit (Qiagen Biosciences, Valencia, CA, USA), following the manufacturer's instructions. 0.3 *μ*g of RNA per sample was reverse transcribed using 25 units of MuLV Reverse Transcriptase (Applied Biosystems, Foster City, CA, USA). 5 *μ*L of the reverse transcription (RT) mixture was utilized in each subsequent qPCR reaction. QPCR was performed using a method described by M. W. Pfaffl for Real-time RT-PCR [18]. This method accounts for effects of amplification efficiency in the following formula:

(1) $$\begin{array}{c} \text{Ratio}=\frac{{\left(E_{\text{target}}\right)}^{\delta \text{Ct}\;\text{target}\;(\text{control}-\text{treated})}}{{\left(E_{\text{ref}}\right)}^{\delta \text{Ct}\;\text{ref}\;(\text{control}-\text{treated})}}. \end{array}$$

Target refers to survivin PCR products, and beta-actin is used as reference to which all survivin PCR products were normalized. *E* refers the amplification efficiency based on the slope of the standard dilution curve. Real-time PCR analysis was performed on the iCycler (Biorad, Hercules, CA, USA) using IQ SYBR Green Supermix (Biorad, Hercules, CA,USA), and survivin PCR primers that were described previously [18]. PCR reaction conditions were: preincubation at 95°C for 10 minutes (1X); denaturation at 95°C for 1 minute, reannealing at 51°C for 30 seconds and extension at 72°C for 30 seconds (40X). Fluorescence emission data were collected at the annealing step. All samples were performed in triplicate in order to calculate statistical significance.

### 2.6. Protein and mRNA Stability Determination

Stability of p62 protein in siRNA transfected cells were described above. To determine p62 protein degradation in the presence of 3-MA, untransfected HT-29 cells were incubated in media containing 5% FBS, and in serum-free media containing vehicle and 10 mM 3-MA for 30 minutes. Then 100 *μ*g/mL CHX was added for the durations indicated in figure, at which time protein extracts were made for western blot and protein quantitation analyses of p62 and beta actin levels in each sample. 

To determine survivin protein half-life in HT-29 cells, 100 *μ*g/mL CHX, which is commonly utilized in numerous publications to inhibit protein synthesis in a wide variety of cultured cells, was also chosen for this study. Cells were treated with this CHX concentration for the durations indicated in figures. To examine the effects of 3-MA on the half-life of survivin, cells that were cultured overnight in serum free media and media containing 5% FBS were pretreated with 10 mM 3-MA for 30 minutes, then CHX was added for the indicated durations. Vehicle + CHX only treatment were included as control. To examine the effects of Atg7 silencing on survivin protein half-life, at 24 hours after transfection with Atg7 siRNA, cells were cultured in serum-free media and media containing 5% FBS overnight, then incubated with CHX for the same durations. Cells transfected with control RNA was included as control. To determine the effects of proteasome inhibitor on survivin half-life, cells were treated with 10 *μ*M MG-132 alone, and 10 mM 3-MA + 10 *μ*M MG-132 for 30 minutes, then with CHX for the indicated durations. Protein extracts were then isolated for western blot analysis and protein quantification as described above. 

To examine the effects of 3-MA on survivin mRNA stability, cells that were cultured overnight in serum-free media and media containing 5% FBS were pretreated with 10 mM 3-MA for 30 minutes, then 1 *μ*M actinomycin D (actD) was added to stop mRNA transcription for 0, 1, 3, 6, and 12 hours. Vehicle + actD only treatment was included as control. To examine the effects of Atg7 silencing on survivin mRNA stability, at 24 hours after transfection with Atg7 siRNA, cells were cultured in serum-free media and media containing 5% FBS overnight, then incubated with actD for the same durations. Cells transfected with control RNA was included as control. At the durations indicated, total RNA was isolated and survivin mRNA stability was determined by realtime quantitative PCR utilizing gene-specific primer as described above.

### 2.7. Statistical Analysis

All data subjected to statistical analysis were repeated in triplicates. Student's *t*-test was used to compare data between two groups. One-way analysis of variance and Bonferroni's correction were used to compare data between three or more groups. *P* value < 0.05 was considered statistically significant. 

## 3. Results

### 3.1. Serum Deprivation Enhanced Induction of Apoptosis in HT-29 Cells by Sulindac Sulfide

**Figure 1:**
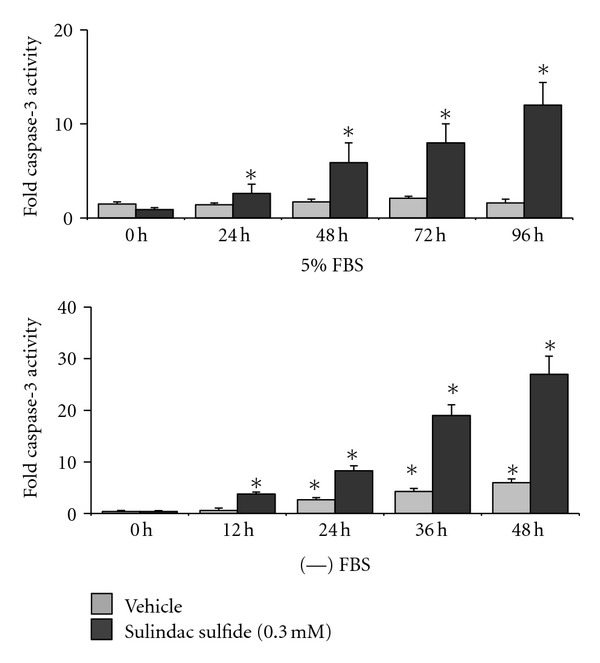
Sulindac sulfide-induced apoptosis is enhanced in serum-free media versus serum-containing media. Equal numbers of HT-29 cells were plated in each well of 6-well plates, allowed to attach overnight, and then incubated in serum-free media and media containing 5% FBS overnight before treatments with vehicle and sulindac sulfide for the indicated durations. Fold apoptosis is determined by measuring caspase-3 activity in total cell lysates. *Indicates significant increase in fold apoptosis relative to 0 hour timepoint, *P* < 0.01.

The extent of apoptosis induction by sulindac sulfide over time in HT-29 cells that were cultured in media containing 5% FBS was compared to that in HT-29 cells that were cultured in media containing no serum. In media containing FBS, vehicle treatment did not increase apoptosis from basal amounts in HT-29 cells for the entire duration of the study (up to 96 hours) (Figure 1). Sulindac sulfide treatment progressively increased apoptosis starting about 24 hours of treatment, to approximately 12 fold by 96 hours of treatment (Figure 1). In media containing no serum, vehicle treatment resulted in low amounts of apoptosis throughout the study duration (Figure 1). Sulindac sulfide treatment progressively increased apoptosis, starting about 12 hours of treatment, to approximately 30-fold by 48 hours of treatment (Figure 1). In support of these data, the annexin V binding assay showed that sulindac sulfide induced 1.5 ± 0.12% apoptotic cells at 0 hours of treatment and 11.6 ± 2.1% apoptotic cells at 96 hours of treatment in serum-containing media, but induced 1.6 ± 0.21% apoptotic cells and 43.5 ± 3.7% apoptotic cells by 48 hours of treatment in serum-free media. Thus, serum deprivation significantly enhanced susceptibility of HT-29 cells to sulindac sulfide-induced apoptosis.

### 3.2. Inhibition of Autophagy Reduced Sulindac Sulfide-Induced Apoptosis in Serum-Free Media, but Facilitated Sulindac Sulfide-Induced Apoptosis in Serum-Containing Media

**Figure 2:**
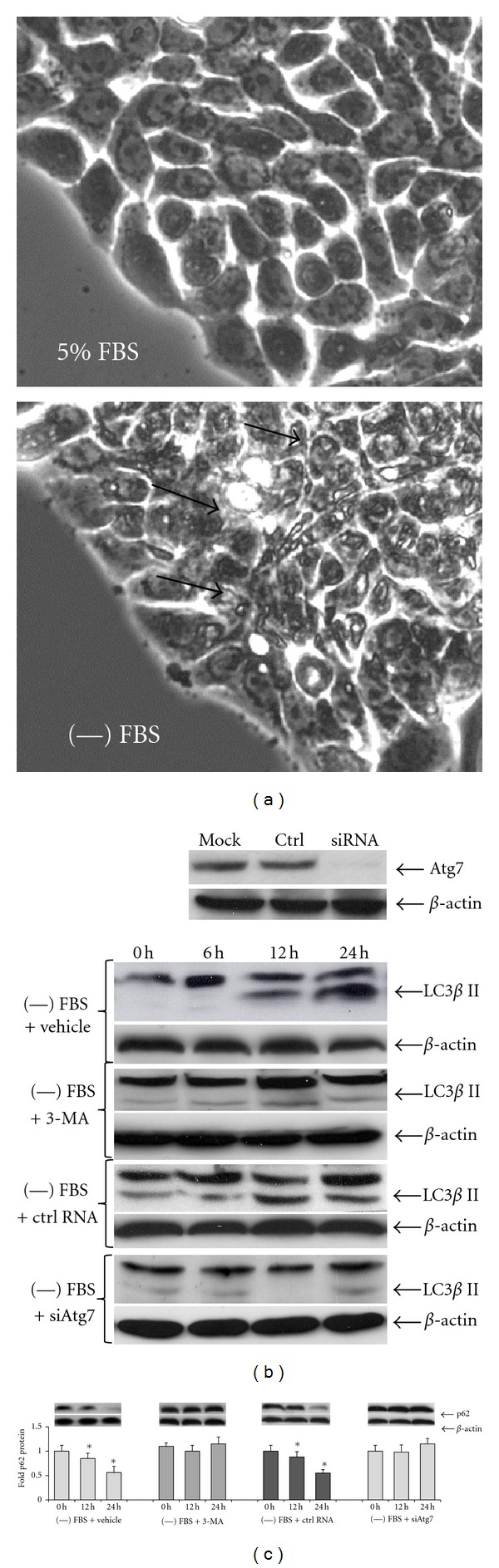
Autophagy is increased in HT-29 cells by serum deprivation and inhibited by 3-methyladenine (3-MA) and Atg7 siRNA. (a) Cells contain basal levels of cytosolic vacuoles in serum-containing media. Cytosolic vacuoles (indicated with arrows) dramatically increased at 24 hours of incubation in serum-free media. (b) Western blots showing silencing of Atg7 by siRNA, but not control RNA (ctrl) relative to mock transfection, conversion of LC3*β* I into LC3*β* II in vehicle and control RNA-treated cells over time, and inhibition of LC3*β* II conversion in 3-MA (10 mM) and Atg7 siRNA (10 ng) treated cells over time in serum-free media. Beta-actin blots show equal protein loading in all samples. (c) Graph of protein quantitations from western blots showing degradation of p62 in vehicle and control RNA treated cells over time, and inhibition of p62 degradation in 3-MA (10 mM) and Atg7 siRNA (10 ng) treated cells over time in serum-free media. One representative western blot out of 3 utilized for the p62 quantitation is shown. *Indicates significantly lower p62 levels relative to 0 hours, *P* < 0.02.

To examine whether or not autophagy is a factor that affects the ability of HT-29 cells to undergo apoptosis in the presence of sulindac sulfide, first autophagy induction was examined in serum free media versus in serum-containing media. In serum-containing media, basal amounts of autophagy occurred in HT-29 cells, as indicated by presence of minimal amounts of cytosolic vacuoles (Figure 2(a)). Addition of 3-MA and transfection of Atg7 siRNA reduced the basal amount of autophagy in serum-containing media. In serum-free media, autophagy was increased in HT-29 cells, as shown by presence of large amounts of cytosolic vacuoles (Figure 2(a)), significant amounts of truncation of the LC3*β* I form (top band of the doublet, Figure 2(b)) into LC3*β* II (bottom band of the doublet, Figure 2(b)), and significant p62 protein degradation over time (Figure 2(c)). Addition of 3-MA to the serum-free media inhibited LC3*β* II conversion and p62 protein degradation (Figures 2(b) and 2(c), resp.). Transfection of control RNA did not reduce Atg7 expression in HT-27 cells (Figure 2(b)), and did not inhibit autophagy induction (Figures 2(b) and 2(c)). In contrast, transfection with Atg7 siRNA reduced Atg7 expression in HT-29 cells (Figure 2(b)) and inhibited autophagy induction (Figures 2(b) and 2(c)).

**Figure 3:**
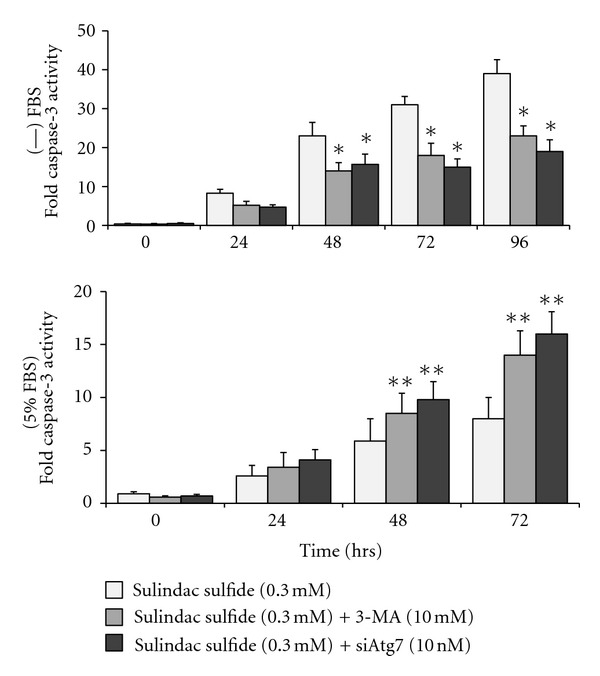
Autophagy inhibition reduced sulindac sulfide-induced apoptosis over time in serum-free media and increased sulfide-induced apoptosis over time in serum-containing media. HT-29 cells that were untransfected and transfected with control RNA and Atg7 siRNA were plated and treated as described in Figure 1. 3-MA was added to cell cultures 30 minutes prior to addition of sulindac sulfide. Apoptosis was determined by measuring fold caspase-3 activity. *Indicates significant reduction in fold caspase-3 activity in cell cultures treated with autophagy inhibitors and sulindac sulfide relative to sulindac sulfide only, *P* < 0.01. **Indicates significant increase in fold caspase-3 activity in cell cultures treated with autophagy inhibitors and sulindac sulfide relative to sulindac sulfide only, *P* < 0.01.

Next, the effect of autophagy inhibition on apoptosis induction by sulindac sulfide in serum free media versus in serum-containing media was examined. In serum-free media, 3-MA treatment and Atg7 siRNA transfection in the presence of sulindac sulfide significantly decreased caspase-3 activation compared to sulindac sulfide only (Figure 3, top graph). In contrast, in serum-containing media, 3-MA treatment and Atg7 siRNA transfection in the presence of sulindac sulfide resulted in significantly increased caspase-3 activation compared to sulindac sulfide only (Figure 3, bottom graph). Taken together, these results indicated that inhibition of autophagy could enhance or reduce sulindac sulfide-induced apoptosis depending on availability of nutrients in the cancer cell's surroundings.

### 3.3. The Extent of Sulindac Sulfide-Induced Apoptosis Corresponds to the Magnitude of Survivin Stability, Which Is Altered by Autophagy Inhibition under Nutrient-Deprived Conditions

**Figure 4:**
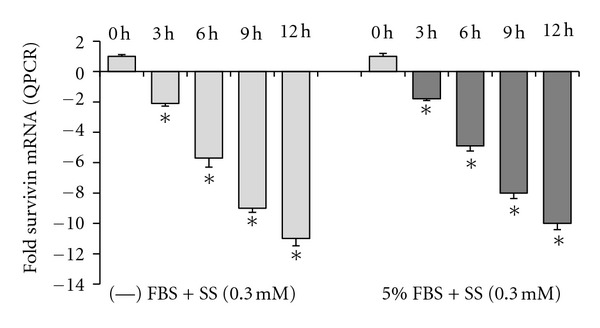
Survivin mRNA down-regulation by sulindac sulfide in serum-free and serum-containing media. Real-time quantitative PCR analysis showed that the rate and extent of downregulation of survivin mRNA levels in the presence of sulindac sulfide were similar over time in serum-free media and serum-containing media. *Indicates significantly reduced survivin mRNA levels relative to 0 hours, *P* < 0.01.

We previously showed that downregulation of the apoptosis inhibitor, survivin, is a key factor in NSAIDs-induced apoptosis in cancer cells, and that sulindac sulfide downregulates survivin mRNA expression [17]. Since sulindac sulfide-induced apoptosis is enhanced in serum-free media versus in serum-containing media (Figure 1), the extent of survivin mRNA downregulation by sulindac sulfide in serum free media versus in serum-containing media was examined. Results showed that sulindac sulfide down-regulated survivin mRNA expression at the same rate, and to similar extent, regardless of serum content in the media (Figure 4). Thus, the extent of sulindac sulfide-induced apoptosis was not due to any differences in the regulation of survivin mRNA expression by sulindac sulfide in serum-free media versus serum-containing media.

**Figure 5:**
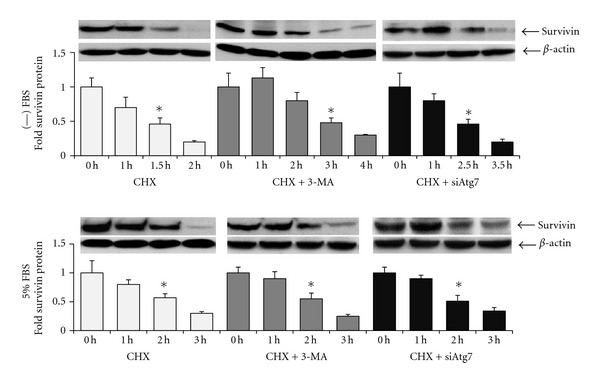
Survivin protein stability is increased in the presence of autophagy inhibitors in serum-free media, but not in serum-containing media. Western blots and protein quantitation graphs showing survivin protein levels over time after addition of CHX (100 *μ*M) to media. One representative western blot out of three repeat experiments was shown for each treatment and condition. The prominent band shown is survivin while the smaller bands are nonspecific, as determined via control experiments utilizing a neutralizing peptide for the survivin antibody. *Indicates the time at which survivin protein is half of the amount at 0 hours (half-life), *P* < 0.02.

To examine whether or not survivin is a factor that affects the ability of HT-29 cells to undergo apoptosis when autophagy is inhibited, survivin mRNA expression and stability, and protein half-life were examined in serum free media versus in serum-containing media in the absence and presence of autophagy inhibitors. 3-MA and Atg7 siRNA did not alter survivin mRNA expression or stability compared to vehicle in serum-free or serum-containing media. In contrast, survivin protein half-life was affected by serum content and autophagy inhibitors: In the presence of cycloheximide (CHX), survivin half-life was approximately 1.5 hours in serum-free media, and approximately 2 hours in serum-containing media (Figure 5). Addition of 3-MA extended survivin protein half-life to approximately 3 hours in serum-free media, but did not alter survivin protein half-life in serum-containing media (Figure 5). Atg7 siRNA also extended survivin protein half-life to approximately 2.5 hours in serum-free media, but not in serum-containing media (Figure 5). 

**Figure 6:**
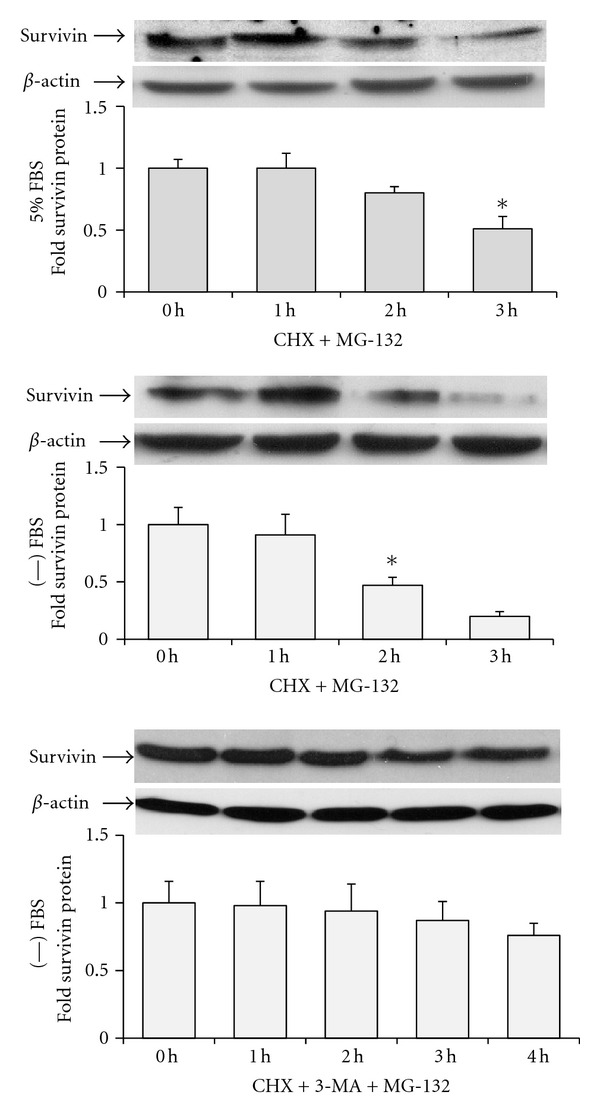
Survivin protein is degraded by ubiquitin proteasome in serum-free and serum-containing media. Western blots and protein quantitation graphs showing that addition of MG-132 proteasome inhibitor (10 *μ*M) extended survivin protein half-life in serum-free and serum-containing media. One representative western blot out of triplicate experiments was shown for each treatment and condition. *Indicates the time at which survivin protein is half of the amount at 0 hours (half-life), *P* < 0.02.

We previously showed that survivin protein is degraded via the ubiquitin proteasome in the presence of NSAIDs such as indomethacin [17]. Therefore, we examined the effect of the proteasome inhibitor MG-132 on survivin protein half-life in the absence and presence of autophagy inhibitors. Results showed that MG-132 alone extended survivin protein half-life to approximately 2 hours in serum-free media, and to approximately 3 hours in serum-containing media (Figure 6). 3-MA in combination with MG-132 extended survivin protein half-life to longer than 4 hours in serum-free media (Figure 6), but did not increase survivin protein half-life beyond 3 hours in serum-containing media. Thus survivin protein is degraded via the proteasome regardless of serum content in the media. However, in serum-free media, regulation of survivin protein degradation appears to be more complex.

## 4. Discussion

HT-29 colon cancer cells were chosen as the model for these studies since (1) we and others showed that sulindac sulfide induces apoptosis in these cells [15, 19]. (2) Autophagy influences the ability of sulindac sulfide to induce apoptosis in these cells [15]. (3) Serum deprivation induces autophagy [20], and our results confirmed that autophagy is measurably increased in HT-29 cells in serum-free media. Thus, HT-29 cells are an appropriate model for examining the influence of autophagy on sulindac sulfide-induced apoptosis under different serum conditions.

Currently, the general view of autophagy is that it is prosurvival, as it can protect cells from prolonged starvation and other stresses, and from treatments with anticancer drugs that are intended to kill cancer cells. A number of published studies indicate that release of cancer cells from this survival mechanism, usually via inhibition of autophagy by inhibitors, can trigger the cells to undergo apoptosis and enhance their susceptibility to anticancer drugs. This was shown in gastrointestinal stromal tumor [21], pancreatic adenocarcinoma [19], and lymphoma [22], among others. A few publications also suggest that autophagy can promote cell death, and that autophagy itself is a form of cell death (reviewed in [9, 23]). Our results indicate that autophagy can support a prosurvival or prodeath outcome for HT-29 colon cancer cells depending on the stress stimuli that they concurrently receive and their physical context. Sulindac sulfide induces significantly greater amounts of apoptosis in HT-29 cells in serum-deprived conditions, where autophagy is significantly increased, versus in conditions where serum is available and autophagy is minimal. One interpretation of these results is that autophagy was not a factor that influenced sulindac sulfide-induced apoptosis in these cells, but something else that was enabled by serum deprivation was. Further investigation showed that there was a clear connection between autophagy and sulindac sulfide-induced apoptosis, since inhibition of autophagy via 3-MA and Atg7 siRNA treatments resulted in reduced ability of sulindac sulfide to induce apoptosis under serum-deprived conditions and enhanced ability of sulindac sulfide to induce apoptosis under conditions where serum is available. 

**Figure 7:**
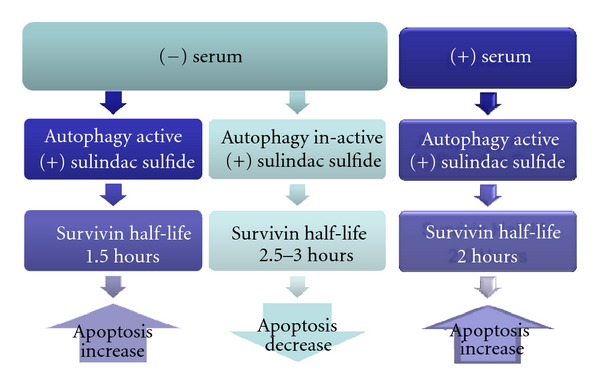
Our model of how autophagy inhibition effects survivin and sulindac sulfide-induced apoptosis in serum-containing and serum-deprived conditions.

The connection between autophagy and other types of cell death, such as apoptosis and necrosis, is poorly understood, and currently a topic of intense research. Our studies showed evidence that autophagy influences apoptosis via common molecular factors that can regulate both processes. These results showed that inhibition of autophagy by 3-MA and Atg7 siRNA had significant effects on the half-life of the apoptosis inhibitor survivin under certain conditions. We previously determined that regulation of survivin levels by NSAIDs is a key factor for apoptosis induction in colon cancer cells [24]. survivin also regulates autophagy [25, 26]. In the present studies, the stability of survivin protein explains the difference in susceptibility of HT-29 cells to sulindac sulfide-induced apoptosis under different conditions. Serum deprivation reduced survivin half-life from about 2 hours to about 1.5 hours, which contributes to the greater potential for apoptosis induction by sulindac sulfide in serum-free media versus in serum-containing media. Inhibition of autophagy in serum-free media increased survivin half-life from about 1.5 hours to about 3 hours, which contributes to resistance to sulindac sulfide-induced apoptosis. In serum-containing media, inhibition of autophagy did not appear to affect survivin half-life. This may have allowed for apoptosis induction in the presence of sulindac sulfide, which downregulates survivin mRNA levels [17, 27]. A diagram representing our model of how autophagy inhibition effects survivin and sulindac sulfide-induced apoptosis is shown in Figure 7.

The mechanism by which autophagy can influence survivin half-life is unclear and requires further investigation. Previously, we showed that survivin protein is degraded by ubiquitin proteasome in the presence of NSAIDs [17]. Here, we showed that proteasome inhibitor MG-132 delayed survivin protein degradation, and 3-MA in combination with MG-132 delayed it even further in serum-free media. These results could mean that survivin protein is degraded via the combination of proteasome and autophagy, or that physical interaction of survivin with another protein when autophagy is inhibited prevents survivin from proteasomal degradation in serum-free media. survivin has been reported to physically interact with Beclin 1 [25], and the molecular network governed by Beclin 1 regulates both autophagy and apoptosis [26]. Whether or not survivin protein stability is influenced by direct physical association with autophagy-associated proteins such as Beclin 1 and/or other proteins yet to be identified remains to be determined.

## 5. Conclusions

Results from the present studies showed that stimulation of autophagy by serum-deprivation enhanced susceptibility to sulindac sulfide-induced apoptosis, whereas basal autophagy under conditions where serum is available suppressed sulindac sulfide-induced apoptosis, in HT-29 colon cancer cells. This indicates that autophagy can support a prosurvival or pro-death outcome for cells depending on the stress stimuli that they receive, and their physical context. Autophagy induction had a significant effect on survivin half-life, and susceptibility of HT-29 cells to sulindac sulfide-induced apoptosis was associated with decreased survivin half-life under various conditions. This indicates that modification of factors, such as survivin, that regulate both the autophagy and apoptosis processes can determine the susceptibility of cancer cells to apoptosis stimuli.
